# IAC方案治疗复发/难治急性髓系白血病疗效与安全性分析：前瞻性随机对照研究

**DOI:** 10.3760/cma.j.issn.0253-2727.2022.04.004

**Published:** 2022-04

**Authors:** 春红 李, 述宁 魏, 少伟 邱, 本法 宫, 晓媛 弓, 艳 李, 云涛 刘, 秋云 房, 广吉 张, 凯奇 刘, 春林 周, 冬 林, 兵城 刘, 迎 王, 营昌 秘, 辉 魏, 建祥 王

**Affiliations:** 中国医学科学院北京协和医学院，中国医学科学院血液病医院（中国医学科学院血液学研究所），细胞生态海河实验室，实验血液学国家重点实验室，国家血液系统疾病临床医学研究中心，天津 300020 Institute of Hematology & Blood Diseases Hospital, Chinese Academy of Medical Sciences & Peking Union Medical College, State Key Laboratory of Experimental Hematology, Haihe Laboratory of Cell Ecosystem, National Clinical Research Center for Blood Diseases, Tianjin 300020, China

**Keywords:** 白血病，髓系，急性, 复发, 难治, 抗肿瘤联合化疗方案, Leukemia, myeloid, acute, Refractory, Relapsed, Antineoplastic combined chemotherapy protocols

## Abstract

**目的:**

评估去甲氧柔红霉素、阿糖胞苷联合环磷酰胺（IAC）方案治疗复发/难治急性髓系白血病（AML）的疗效和安全性。

**方法:**

研究设计为前瞻性随机对照临床试验。纳入2016年7月1日至2019年10月9日于中国医学科学院血液病医院确诊的除急性早幼粒细胞白血病以外的复发/难治AML患者。入组患者进行分层随机分组，试验组选择IAC方案再诱导治疗，对照组的治疗方案由医师根据经验从多个再诱导治疗方案中选择。符合条件患者在挽救治疗后尽可能进行异基因造血干细胞移植（allo-HSCT)。分析两组的疗效及安全性。

**结果:**

42例患者入组，IAC 组22例，对照组20例，中位年龄为36（15～58）岁。①两组患者总有效率（ORR）分别为 71.4％和40.0％（*P*＝0.062）；完全缓解（CR）率分别为66.7％和40.0％（*P*＝0.121）。中位随访时间为10.5（1.7～32.8）个月，IAC组中位总生存（OS）时间为14.1（0.6～49.1）个月，对照组为9.9（2.0～53.8）个月（*P*＝0.305）。1年OS率两组分别为54.5％（95％ *CI* 33.7％～75.3％）和48.2％（95％ *CI* 25.9％～70.5％），差异无统计学意义（*P*＝0.305）。②不良反应主要表现为骨髓抑制导致的血细胞减少。IAC组和对照组3～4级血液学不良反应发生率分别为100％（22/22）和95％（19/20）。化疗后粒细胞缺乏持续的中位时间分别为20（8～30）d和14（5～50）d（*P*＝0.023)。③早期复发（12个月内复发）组CR率为46.7％，晚期复发（12个月后复发）组CR率为72.7％（*P*＝0.173），两组中位OS时间分别为9.9（1.7～53.8）个月和19.3（0.6～40.8）个月（*P*＝0.420），1年OS率分别为45.3％（95％ *CI* 27.2％～63.3％）和66.7％（95％ *CI* 40.0％～93.4％）（*P*＝0.420），差异均无统计学意义。④对于挽救治疗后的患者，根据是否接受allo-HSCT分组，移植组和未移植组1年OS率分别为87.5％（95％ *CI* 71.2％～100％）和6.3％（95％ *CI* 5.7％～18.3％），移植组明显高于未移植组（*P*<0.001）。

**结论:**

IAC方案与根据经验选择的方案对复发/难治AML患者具有相似的疗效及安全性，复发/难治患者应及早进行allo-HSCT，以期获得长期生存。

## Abstract (en)

**Objective:**

To evaluate the efficacy and toxicity profiles of idarubicin, cytarabine, and cyclophosphamide (IAC) in relapse/refractory acute myeloid leukemia (AML).

**Methods:**

This study was a prospective, randomized controlled clinical trial with the registration number NCT02937662. The patients were randomly divided into two groups. The experimental group was treated with an IAC regimen, and the regimen of the control group was selected by doctors according to medication experience. After salvage chemotherapy, allogeneic hematopoietic stem cell transplantation (allo-HSCT) was conducted as far as possible according to the situation of the patients. We aimed to observe the efficacy, safety, and toxicity of the IAC regimen in relapse/refractory AML and to explore which is the better regimen.

**Results:**

Forty-two patients were enrolled in the clinical trial, with a median age of 36 years (IAC group, 22 cases and control groups, 20 cases). ①The objective response rate was 71.4％ in the IAC group and 40.0％ in the control group (*P*＝0.062); the complete remission (CR) rate was 66.7％ in the IAC group and 40.0％ in the control group (*P*＝0.121). The median follow-up time of surviving patients was 10.5 (range: 1.7–32.8) months; the median overall survival (OS) was 14.1 (range: 0.6–49.1) months in the IAC group and 9.9 (range: 2.0–53.8) months in the control group (*P*＝0.305). The 1-year OS was 54.5％ (95％ *CI* 33.7％–75.3％) in the IAC group and 48.2％ (95％ *CI* 25.9％–70.5％) in the control group (*P*＝0.305), with no significant difference between these two regimens. ②The main hematologic adverse events (AEs) were anemia, thrombocytopenia, and neutropenia. The incidence of grade 3–4 hematologic AEs in the two groups was 100％ (22/22) in the IAC group and 95％ (19/20) in the control group. The median time of neutropenia after chemotherapy in the IAC group and control group was 20 (IQR: 8–30) and 14 (IQR: 5–50) days, respectively (*P*＝0.023). ③The CR rate of the early relapse (relapse within 12 months) group was 46.7％ and that of the late relapse (relapse after 12 months) group was 72.7％ (*P*＝0.17). The median OS time of early recurrence was 9.9 (range:1.7–53.8) months, and that of late recurrence patients was 19.3 (range: 0.6–40.8) months (*P*＝0.420), with no significant differences between the two groups. The 1-year OS rates were 45.3％ (95％ *CI* 27.2％–63.3％) and 66.7％ (95％ *CI* 40.0％–93.4％), respectively (*P*＝0.420). Survival analysis showed that the 1-year OS rates of the hematopoietic stem cell transplantation group and non-hematopoietic stem cell transplantation group were 87.5％ (95％ *CI* 71.2％–100％) and 6.3％ (95％ *CI* 5.7％–18.3％), respectively. The OS rate of the hematopoietic stem cell transplantation group was significantly higher than that of the non-hematopoietic stem cell transplantation group (*P*<0.001).

**Conclusion:**

The IAC regimen is a well-tolerated and effective regimen in relapsed/refractory AML; this regimen had similar efficacy and safety with the regimen selected according to the doctor's experience for treating relapsed/refractory AML. For relapsed/refractory patients with AML, allogeneic hematopoietic stem cell transplantation should be attempted as soon as possible to achieve long-term survival.

目前化疗治疗急性髓系白血病（AML）的完全缓解（CR）率为60％～80％[1]–[3]，有20％以上的患者很难达到CR，另外对于获得缓解的AML患者，约50％会出现复发[4]。目前尚无统一的高效治疗方案用于复发/难治AML患者的治疗。烷基化剂环磷酰胺（CTX）是细胞周期非特异性药物，作用于细胞静息期，在体内经肝细胞色素P450酶和化学活化，代谢产物磷酰胺氮芥产生链间和链内DNA交联，产生细胞毒性作用[5]。1990年Brown等[6]将大剂量依托泊苷联合CTX应用于复发和难治性急性白血病和淋巴瘤的挽救治疗，35％的患者获得CR。也有小病例系列或单臂研究结果显示，大剂量依托泊苷联合CTX作为原发难治性或复发性AML的挽救治疗，CR率为28％～57％[7]–[9]。近年来我中心进行了含CTX的MAC方案［米托蒽醌+阿糖胞苷（Ara-C）+CTX］治疗复发AML患者的临床试验，初步的结果提示MAC方案对复发白血病具有良好的疗效[10]。因而我们设计了本项研究，进一步观察含CTX的IAC方案的疗效及安全性。

## 病例与方法

1. 病例：本研究为前瞻性随机对照临床试验，采用分层随机方法。将受试者依据难治AML和非难治的复发AML分层，然后随机分入试验组和对照组，难治AML包括原发难治及早期复发患者（第1次CR期<12个月）；非难治的复发AML主要包括晚期复发（第1次CR期≥12个月）患者。研究方案经本单位伦理委员会批准（IIT2016006-EC-1）。入组患者自愿加入临床试验并签署知情同意书。

入选标准：①纳入2016年7月1日至2019年10月9日确诊的除急性早幼粒细胞白血病以外的复发/难治AML患者，患者符合复发/难治诊断标准[11]，分型诊断参照法英美协作组诊断标准（FAB标准）和2008年WHO AML诊断和分类标准[12]。②复发为骨髓细胞形态学复发。③年龄≥15岁且<60岁。④美国东部肿瘤协作组体力状态评估（ECOG-PS）为0～2分。排除标准：①既往曾接受过包含CTX的再诱导治疗方案；②伴有其他血液系统疾病或活动性心脏疾病；③既往曾接受过异基因造血干细胞移植（allo-HSCT）治疗或伴有BCR-ABL融合基因需要接受酪氨酸激酶抑制剂（TKI）治疗的患者；④单纯髓外白血病复发；⑤WHO AML分类属于不另做分类的AML的亚类中急性全髓增殖症伴骨髓纤维化及髓系肉瘤患者。

2. 治疗方案：IAC方案具体用药：去甲氧柔红霉素（IDA）8 mg·m^−2^·d^−1^，第1～3天；Ara-C 100 mg·m^−2^·d^−1^，第1～7天；CTX 350 mg·m^−2^·d^−1^，第2、5天。对照组用药：研究者根据经验从下述的治疗方案中选择。FLA±G方案:氟达拉滨（Flu）30 mg·m^−2^·d^−1^，第1～5天；Ara-C 2 g·m^−2^·d^−1^，第1～5天；可同时应用G-CSF。DA方案：Ara-C 100 mg·m^−2^·d^−1^，第1～7天；柔红霉素（DNR）60 mg·m^−2^·d^−1^，第1～3天。地西他滨+AA方案：地西他滨20 mg·m^−2^·d^−1^第1～5天（可根据情况缩短，但不少于3 d）；阿克拉霉素20 mg/d，第4～8天；Ara-C 100 mg/d，第4～8天（可根据情况延长，最长至第10天）。AAG方案：阿克拉霉素20 mg/d，第1～7天；Ara-C 100 mg/d，第1～10天（可根据情况延长，最长至第14天）；可同时应用G-CSF。在诱导治疗达CR后尽可能选择allo-HSCT。对于未能进行allo-HSCT的患者，医师可以根据经验选择缓解后巩固治疗方案。

3. 疗效评价：研究主要终点：CR率。次要终点：诱导治疗相关死亡率、总生存（OS）及化疗相关不良反应。疗效分为CR、部分缓解（PR）和未缓解（NR），参考张之南主编的《血液病诊断及疗效标准》（第三版）评估。CR标准：骨髓原始细胞<5％，外周血中性粒细胞绝对计数≥1.0×10^9^/L，PLT≥100×10^9^/L，没有髓外浸润；PR标准：骨髓原始细胞5％～25％（下降至少50％），外周血符合CR标准；治疗失败即NR。总有效率（ORR）为CR率、PR率之和。

4. 安全性评价：记录患者在接受化疗后出现的不良反应，包括外周血细胞计数、发热及感染情况。并统计患者在接受化疗后粒细胞缺乏（粒缺）持续的时间。不良事件的报告和分级参考NCI常见不良事件术语标准（NCI CTC AE）4.0版。

5. 随访：随访截止日期为2021年10月11日。总生存（OS）时间定义为开始接受挽救治疗至死亡或随访终止的时间。生存患者的中位随访时间为10.5（1.7～32.8）个月。

6. 统计学处理：应用SPSS 26.0软件进行统计学分析。计量资料的组间比较采用*t*检验，当组间计量资料不符合正态分布或组间计量资料方差不齐时采用Mann-Whitney *U*秩和检验，分类变量的组间比较采用*χ*^2^检验，当不满足*χ*^2^检验时应用Fisher检验，生存率的比较采用Log-rank检验，生存曲线绘制采用Kaplan-Meier法。*P*<0.05为差异有统计学意义。

## 结果

1. 患者的临床特征：42例患者符合本试验入排标准，IAC 组22例，对照组20例。男18例，女24例，患者的中位年龄为36（15～58）岁，入组时疾病状态：原发难治患者1例，早期复发患者26例，晚期复发患者15例，两组患者的临床特征差异无统计学意义（表1）。

**表1:** 42例复发/难治急性髓系白血病患者的一般临床特征

临床特征	IAC组	对照组	统计量	*P*值
性别（例，男/女）	9/13	9/11	0.07	1.000
年龄［岁，*M*（范围）］	36（18～52）	36（15～58）	0.27	0.870
ELN预后分组［例（％）］				
预后良好	13（59.1）	8（40.0）	1.59	0.452
预后中等	4（18.2）	6（30.0）		
预后不良	5（22.7）	6（30.0）		
遗传学改变［例（％）］				
FLT3-ITD	1（4.5）	2（10.0）	0.47	0.598
CBFβ-MYH11	3（13.6）	0（0）	2.94	0.233
RUNX1-RUNX1T1	6（27.3）	3（15.0）	0.94	0.460
CEBPA双突变	4（18.2）	5（25.0）	0.29	0.714
NPM1突变	5（22.7）	4（20.0）	0.05	1.000
ASXL1突变	3（13.6）	1（5.0）	0.91	1.608
WBC［×10^9^/L，*M*（*P*_25_，*P*_75_）］	20.1（4.9,57.0）	23.9（11.5,96.9）	0.79	0.374
疾病状态（例，早期复发/晚期复发/原发难治）	13/8/1	14/6/0	1.22	0.544
既往接受化疗次数［*M*（*P*_25_，*P*_75_）］	5（4,6）	5（2,8）	0.40	0.526

注：IAC：去甲氧柔红霉素+阿糖胞苷+环磷酰胺；ELN：欧洲白血病网

2. 疗效和生存分析：所有患者挽救治疗CR率为52.4％，中位OS时间为12.7（0.6～53.8）个月。两组患者的疗效比较见表2。ORR、CR率、OS时间在两组中差异均无统计学意义（*P*值均>0.005）。两组的OS曲线见图1。

**表2:** IAC组和对照组患者疗效比较

组别	例数	ORR［例（％）］	CR［例（％）］	死亡［例（％）］	接受allo-HSCT［例（％）］	1年OS率（％）（95％ *CI*）	OS时间［月，*M*（范围）］
IAC组	22	15（71.4）	14（66.7）	11（50.0）	9（42.9）	54.5（33.7～75.3）	14.1（0.6～49.1）
对照组	18	8（40.0）	8（40.0）	13（65.0）	8（40.0）	48.2（25.9～70.5）	9.9（2.0～53.8）

统计量		4.11	2.92	0.96	0.03	1.00	1.00
*P*值		0.062	0.121	0.366	1.000	0.305	0.305

注：IAC：去甲氧柔红霉素+阿糖胞苷+环磷酰胺；ORR：总反应率；CR：完全缓解；OS：总生存；allo-HSCT：异基因造血干细胞移植

**图1:**
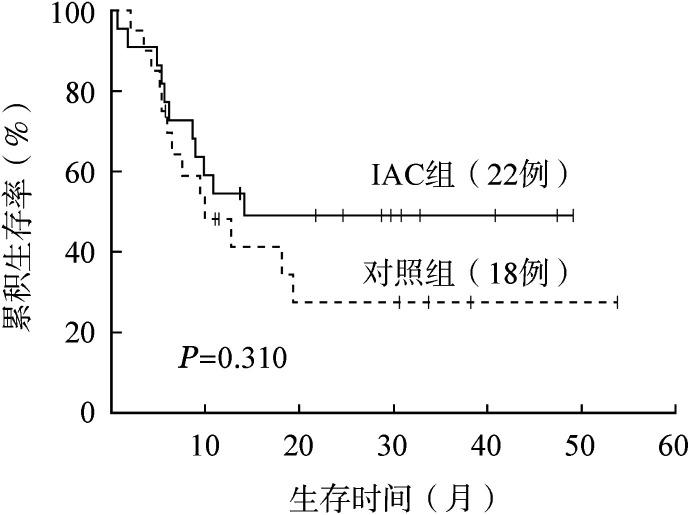
两种方案对复发/难治急性髓系白血病患者总生存的影响 IAC：去甲氧柔红霉素+阿糖胞苷+环磷酰胺

诱导治疗后，42例患者中共17例（34.1％）患者进行allo-HSCT，移植前患者疾病状态：CR 9例，NR 7例，PR 1例。移植组1年OS率为87.5％（95％ *CI* 71.2％～100％），未移植组为6.3％（95％ *CI* 5.7％～18.3％），移植组生存明显优于未移植组（*P*<0.001）（图2）。

**图2:**
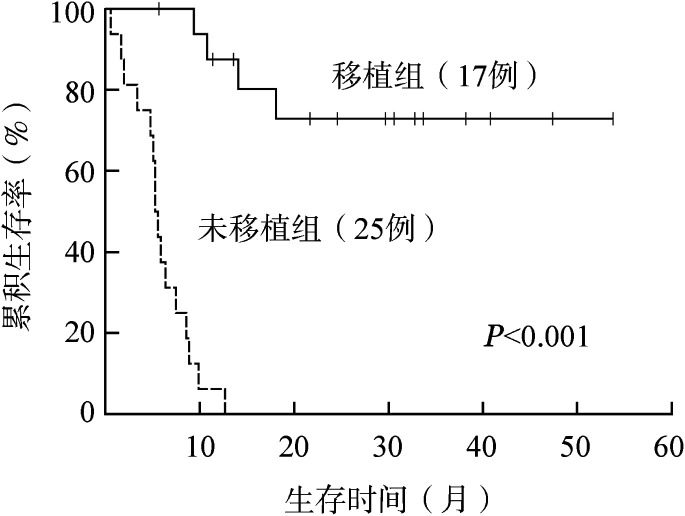
异基因造血干细胞移植对复发/难治急性髓系白血病患者总生存的影响

根据第1次CR至复发的时间间隔，将患者分为早期复发组（30例）和晚期复发组（11例）。患者对再次诱导治疗的CR率，早期复发组为46.7％（14/30），晚期复发组为72.7％（8/11，*P*＝0.173），早期复发组中位OS时间为9.9（1.7～53.8）个月，晚期复发组中位OS时间为19.3（0.6～40.8）个月（*P*＝0.420），差异均无统计学意义。两组1年OS率分别为45.3％（95％ *CI* 27.2％～63.3％）和66.7％（95％ *CI* 40.0％～93.4％）（*P*＝0.420）（图3）。

**图3:**
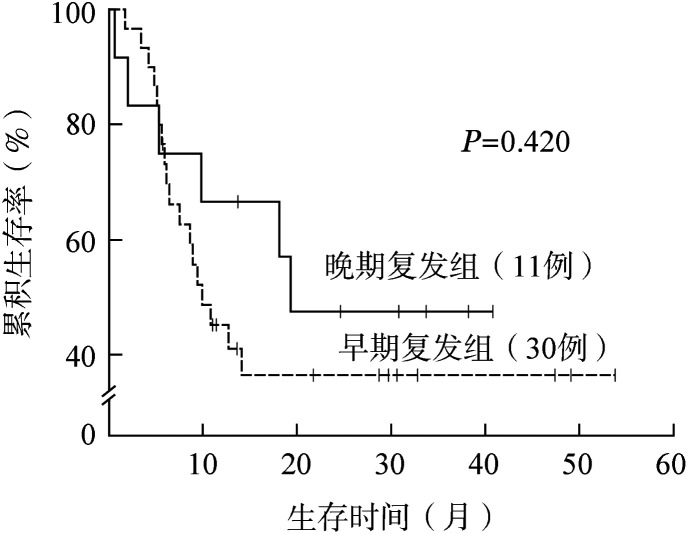
早期复发和晚期复发急性髓系白血病患者的总生存曲线

3. 安全性：IAC组和对照组各有1例患者30 d内死亡，30 d内死亡率差异无统计学意义（*P*＝0.730），IAC组1例患者死于化疗后骨髓抑制期血流感染，对照组1例患者死于骨髓抑制期肠梗阻合并血流感染。

IAC组和对照组患者的不良反应主要为血小板减少、粒细胞缺乏及贫血的Ⅲ～Ⅳ度血液学不良反应，发生率分别为100％和95.0％（*P*＝0.476）。IAC组和对照组均有2例患者发生血流感染，发生率分别为9.1％和10.0％（*P*＝1.000），各有1例患者发生肠梗阻（*P*＝1.000）。两组粒缺伴发热发生率分别为90.9％和80.0％（*P*＝0.400），化疗后中位粒缺持续时间分别为20（8～30）d和14（5～50）d，前者明显长于后者（*P*＝0.023）。

## 讨论

综合国内外AML治疗指南，复发/难治性AML患者的治疗首先推荐参加临床试验。治疗的总原则包括：使用无交叉耐药的新药组成的新联合化疗方案；应用新的靶向治疗药物；含中、大剂量Ara-C的联合方案；60岁以下、缓解期≥12个月复发者可以用原诱导治疗方案再诱导。复发难治患者在取得CR后，应尽可能选择allo-HSCT[11],[13]。以化疗药物为主的挽救治疗的重要组成部分包括蒽环类和大剂量Ara-C，通常将嘌呤类似物（如Flu或克拉屈滨）或拓扑异构酶Ⅱ抑制剂依托泊苷与该方案联合[14]。国内外研究设计了一系列非交叉耐药的、以Ara-C为基础的联合化疗方案，如FLAG和FLAG-IDA方案，CR率为50％～60％[15]–[17]。

本研究中IAC组和对照组CR率差异无统计学意义。IAC方案的CR率66.7％和国外报道的用于成人复发/难治AML治疗的一系列方案，包括以Ara-C为主的方案、非Ara-C联合方案、低甲基化药物为主的方案和单一新药物等方案的疗效相当[18]–[19]。IAC组中位OS时间为14.1个月，虽长于对照组的9.9个月，但差异无统计学意义，该结果也与既往其他研究相似，即对于复发/难治AML患者，虽然选择不同的方案，但均显示出相似的治疗效果[17]。总体而言，IAC组与对照组疗效相当，提示对于复发/难治AML患者，IAC方案也可以作为挽救治疗方案，本方案为复发/难治AML患者增加一种治疗选择。挽救治疗的目的是为患者争取移植机会，本研究中的未移植组患者生存期最长为12个月，挽救治疗后，移植组的生存时间明显长于未移植组，提示复发患者只有接受移植才有可能获得长期生存。中国复发难治AML诊疗指南（2021年版）建议：复发/难治AML患者获得缓解后，在条件许可情况下应尽早进行allo-HSCT。对于某些患者，尤其是原发耐药或早期复发且预估缓解率非常低的患者也可以直接采取allo-HSCT作为挽救治疗措施[11]。

本研究中两组患者最常见的不良反应为骨髓抑制及感染，绝大部分患者可以恢复正常造血，个别患者因重症感染死亡，早期死亡率两组差异无统计学意义。IAC组患者粒缺持续时间较对照组更长，提示该联合方案对骨髓的抑制作用更强。粒缺期延长将增加感染的风险，但我们没有发现试验组重症感染风险增加，感染导致的死亡率也无升高，可能与入组患者年龄<60岁有关，IAC方案对老年难治/复发AML患者的疗效及不良反应仍然需进一步研究探讨。

对于年龄≤60岁的AML复发患者，欧洲白血病网（ELN）将从缓解到复发的时间间隔作为影响患者预后的因素之一[20]，本研究结果提示晚期复发组的挽救治疗CR率高于早期复发患者，远期疗效优于早期复发患者，但差异均无统计学意义，可能与样本量小有关。

本研究纳入的病例数有限，结论需进一步增加研究样本量进行验证。目前国内外指南均推荐将新的靶向药物应用于初诊AML及复发/难治AML的治疗[11],[21]。今后我们会进一步开展靶向药物联合IAC方案的临床研究。

综上，IAC方案及对于复发/难治 AML 患者具一定的疗效和安全性，复发/难治的成人AML患者可考虑将其作为移植前的挽救治疗。对于可以应用靶向药物治疗的患者，可以选择联用靶向药物治疗。获得缓解后的复发/难治患者应尽早进行allo-HSCT，以获得长期生存的可能。
